# Suppression of Tumorigenicity 5 Ameliorates Tumor Characteristics of Invasive Breast Cancer Cells *via* ERK/JNK Pathway

**DOI:** 10.3389/fonc.2021.621500

**Published:** 2021-07-28

**Authors:** Jianghong Cheng, Mingli Li, Chi-Meng Tzeng, Xingchun Gou, Shuai Chen

**Affiliations:** ^1^Shaanxi Key Laboratory of Brain Disorders and School of Basic Medical Science, Xi’an Medical University, Xi’an, China; ^2^Translational Medicine Research Center (TMRC), School of Pharmaceutical Science, Xiamen University, Xiamen, China; ^3^Department of Otolaryngology-Head and Neck Surgery, The First Affiliated Hospital of Xiamen University, Xiamen, China; ^4^Xiamen Key Laboratory of Otolaryngology Head and Neck Surgery, Xiamen, China; ^5^Institute of Basic and Translational Medicine, Xi’an Medical University, Xi’an, China; ^6^Academician Workstation of Chen Zhi-nan, Xi’an Medical University, Xi’an, China

**Keywords:** suppression of tumorigenicity 5, ERK1/2, JNK, methylation, breast cancer

## Abstract

**Background:**

Suppression of tumorigenicity 5 (ST5) has been considered as a tumor suppressor gene in HeLa tumor cells. However, its role in the progression of breast cancer remains vague.

**Methods:**

Online database analysis was determined by Oncomine and Breast Cancer Gene-Expression Miner v4.4 (bc-GenExMiner v4.4). Tumor biology behaviors were measured by MTT assay, wound healing model, Transwell and Flow cytometry assays. Methylation-specific PCR (MSP) was employed to detect promoter methylation.

**Results:**

Low level of ST5 was observed in breast cancer specimens, particularly in recurrent, invasive breast cancer cases compared to para-carcinoma tissue or non-invasive breast cancer. The downregulation of ST5 was also proved in MDA-MB-231 and SKBR3 cell lines with a high invasive capability as compared to MCF-7 cell with a low invasive capability. ST5 was negatively associated with pathological stages of breast cancer. ST5-downregulation promoted, while ST5-upregulation inhibited the progression of cell proliferation, cell cycle and migration of MDA-MB-231 cells. Additionally, ST5 knockdown inhibited, whereas ST5 overexpression promoted apoptosis of MDA-MB-231 cells. However, ST5 modification, either upregulation or downregulation, had no significant impact on tumor behaviors of MCF-7 cells. Mechanistically, ST5 protein ablation activated, while ST5-upregulation repressed the activities of phosphorylated ERK1/2 and JNK, and subsequently the expression of c-Myc. PD98059-mediated ERK1/2 inhibition abolished the stimulatory effects of ST5-depletion on ERK1/2/JNK/c-Myc signaling axis, and ST5 depletion-mediated cell over-proliferation and migration. Of note, ST5 reduction in invasive breast cancer cells should implicate in the hypermethylation of *ST5* promoter region.

**Conclusion:**

Our findings suggest that ST5 potentially acts as a tumor suppressor gene in invasive breast cancer through regulating ERK/JNK signaling pathway and provide a novel insight for breast cancer treatment.

## Introduction

Breast cancer is the leading cause of cancer deaths among women worldwide (1). According to the statistics, more than one million women are diagnosed with breast cancer every year in the world (2). Based on the status of estrogen receptor (ER), progesterone receptor (PR) and receptor tyrosine-protein kinase erbB-2 (HER2), breast cancer is classified as Luminal A, Luminal B, HER2-positive (HER2-E) and triple-negative breast cancer (TNBC) subtypes (3). Although the endocrine therapy is the preferred treatment and has a better therapeutic effect for hormone receptor-positive breast cancer, quite a few hormone receptor-positive patients will develop either primary or secondary drug resistance (4). Additionally, most metastatic breast tumor patients, such as patients with TNBC subtype, has poor prognosis and high recurrence rate (5). Currently, there remains plenty of challenges for treating highly malignant and invasive breast cancer due to lacking of well-defined molecular targets.

Suppression of tumorigenicity 5 (ST5), also known as DENND2B, is a member of DENN protein family which is localized to chromosome 11p15.2 (6). DENN protein family presents significantly differential expression between normal and tumor cells (6–8). Actually, accumulating evidence indicate that ST5 is a tumor suppressor (9, 10). *ST5* gene can encode cleavage ST5 protein P70 (70kD) and P82 (82kD), and the full length of ST5 protein (126kD, P126) (11). In comparison to normal ovarian epithelial cells, ST5 is down-regulated in ovarian carcinoma cells, which upregulation notably inhibited tumor cells growth (12). Exogenous ST5 suppresses tumor formation and growth of Hela cells-bearing mice *in vivo* (9), and this inhibition effect is correlated with the elevation of cleavage ST5 (P70), but not P126 (13). Moreover, combined with microarray gene chip study, the significant elevation of ST5 (P126kD) in uterine leiomyoma is observed compared to that in normal myometrium (14). Therefore, abnormal expression of ST5 exists in a variety of tumor types. DNA promoter methylation profiles are the most common epigenetic modification of tumor suppressors in breast cancer (15). Actually, hypermethylation of DENN family was observed in several tumor types, which facilitates the tumor progression (16, 17). However, the methylation state of *ST5* promoter region and expression pattern of ST5 in breast cancer remains vague.

The full length ST5 protein (P126) stimulates mitogen-activated protein (MAP) kinase activation in response to epidermal growth factor (EGF) (18). ST5 can activate Ras/ERK signaling in the presence of EGF, contributing to β-cell proliferation (19). A multi-domain adaptor protein intersectin-s can integrate DENND2B (ST5) to promote the recycling of ligand-free epidermal growth factor receptor (EGFR) to the cell surface that is a reoccurring theme in cancer cell growth (20). It is also confirmed that ST5 could interact with Rab13, leading to the promotion on migration, invasion and tumorigenicity phenotype of normal breast epithelial cells MCF-10A (21). The results suggest that ST5 may be involved in the regulation of tumor behavior processes such as proliferation and metastasis in gynecological oncology, but the specific function and the underlying mechanism of ST5 in breast cancer still need more deeply investigations.

Herein, we analyzed the expression pattern of ST5 in breast cancer specimens and explored its biological function in breast cancer cells with a low-invasive or high-invasive capability. ST5-mediated the downstream signaling transduction was also determined. Additionally, we investigated the association between ST5 expression and promoter methylation in breast cancer cells with high-invasive capability. Collectively, our data illustrate that ST5 potentially acts as a potent tumor suppressor in the metastatic progression of breast cancer, but had a slight impact on tumor behaviors in breast cancer cells with a low-invasive capability.

## Materials and Methods

### Online Database Analysis

This work was approved by internal review board for ethical issues of the First Affiliated Hospital of Xiamen University. The differential expression of ST5 in different subtypes of breast cancer tissues, and the association between ST5 status and tumor stage (SBR standard classification), were determined using the Breast Cancer Gene-Expression Miner v4.4 (bc-GenExMiner v4.4). The expression of ST5 in patients with no recurrence and recurrence, patients with ductal breast cancer and invasive breast cancer, was assessed by using Oncomine database.

### Cell Culture and Treatment

The normal mammary epithelial cell line (MCF-10A), ER-positive breast cancer cell (MCF-7), adriamycin resistant MCF-7 cells (MCF-7/ADR), ER-negative cell line (MDA-MB-231), and ER/PR-positive and Her2-negative breast cancer cell lines (SKBR3) were purchased from American Type Culture Collection (ATCC). MCF-10A cells were cultured in Dulbecco’s modified eagle medium (DMEM)/F12 (1:1) medium supplemented with 5% horse serum (16050-122; Invitrogen, USA), 10 μg/ml insulin (I-1882; Sigma, USA), 20 ng/ml epidermal growth factor (EGF; SRP3027; Sigma, USA), 100 ng/ml cholera toxin (C-8052; Sigma, USA), 0.5 μg/ml hydrocortisone (H-0888; Sigma, USA). MDA-MB-231 and SKBR3 cells were incubated with RPMI 1640 (SH30809.01; Hy Clone, USA) and MCF-7 were cultured in Dulbecco’s modified eagle medium (DMEM) (SH30022.01; HyClone, USA) and supplemented with 10% fetal bovine serum (FBS; SH30070.03; HyClone, USA). MCF-7/ADR cells were maintained in RPMI-1640 medium supplemented with 10% FBS and 1 μM doxorubicin. All cells were incubated in a humidified incubator containing 5% CO2 at 37°C. For the treatment of methylation inhibitor, MDA-MB-231 cells were exposed to 10 μM and 20 μM of 5-aza-2’-deoxycytidine (5-Aza) (Sigma-Aldrich, USA) in DMSO or DMSO alone for 72 h. For the treatment of MEK inhibitor, siST5-transfected MDA-MB-231 cells were exposed to 10 μM ERK inhibitors (PD98059, HY-12028, MCE, US) for 24 h. After the initial 5-aza-dC treatment or PD98059, cells were subjected to the subsequent experiments.

### Cell Transfection

Briefly, the CDS fragment of ST5 (126kD, P126) was inserted into pEGFP-C1 plasmid. Control pEGFP-C1 vector and recombinant pEGFP-C1-ST5 plasmids were transfected into MDA-MB-231 and MCF-7 cells using the TurboFect transfection reagent (R0531; Thermo Fisher Scientific, USA). At 24 h post-transfection, cells were harvested for further investigation. For RNA interfere (RNAi)-mediated depletion of ST5, cells were transfected with negative control siRNA (siNC) or siST5 (sequence) using the TurboFect transfection reagent. At 72 h post-transfection, cells were used for subsequent experiments.

### Human Specimens and Immunohistochemical Staining

Human breast cancer tissues were collected from the First Affiliated Hospital of Xiamen University. Clinicopathological characteristics of Breast Cancer patients were presented in **Table 1**. Human breast cancer tissue chips or purchased from OUTDO BIOTECH (Shanghai, China). A signed informed consent form was obtained from all subjects, and this study was approved by the ethics committee of the First Affiliated Hospital of Xiamen University (2016XMU-BIO-77). Tissue chips and tissue sections were dewaxed in xylene solution, and hydrated in graded ethanol solutions. After antigen retrieval using high pressure method, the sections were incubated with 0.3% H_2_O_2_ for 20 min. Then, sections were incubated overnight with a primary antibody against ST5 (ab187759, Abcam, UK). The next day, the sections were hybridized with the secondary antibody and subjected to chromogenic reaction using DAB reagent (1:50). The staining intensity and the number of positive cells in the tissue chip, which were analyzed using the Image Pro Plus software v6.0 (Media Cybernetics, Inc., Maryland, USA) (negative: –; weakly positive: +, <20%; middling positive: ++, 20%–50%; strongly positive: +++, >50%). Tumors were categorized as “Low” or “High” grade based on relative ST5 expression according to the staining intensity (++ and +++ were defined as High, - and + were defined as Low). Relative staining intensity was evaluated by three independent pathologists.

**Table 1:** Chi-squared analysis of contingency tables between ST5 status and clinicopathological characteristics of Breast Cancer patients.

Characteristics	Relative ST5 expression		*P value*
	Low (n = 33)	High (n = 26)	
**Gender**			0.4407
Male	0	1	
Female	33	25	
**Age**			0.4096
≤50	9	10	
>50	24	16	
**Tumor grade**			0.0397*
G1	2	8	
G2	12	8	
G3	19	10	
**Tumor diameter (cm)**			0.3716
≤5	29	25	
>5	4	1	

*P < 0.05 was considered statistically significant.

### MTT Assay

MDA-MB-231 and MCF-7 cells transfected with pEGFP-C1 vector, recombinant pEGFP-C1-ST5 and small interfering RNAs were seeded into 96-well plates at a density of 2 x 10^3^ cells/well, and cultured for additional 24 h, 48 h and 72 h. After incubation, 10 μL MTT (Sigma-Aldrich, USA) was added to the medium and incubated for another 4 h. Then, OD values were measured at the wavelength of 492 nm using a Multiscan plate reader (MK3, Thermo, USA).

### Flow Cytometry

All group cells were seeded in 6-well plates at a density of 5 × 10^5^ cells/well. For cell cycle detection, cells were digested with trypsin without Ethylene Diamine Tetraacetic Acid (EDTA). After fixing with 1ml 70% ethanol overnight, cells were centrifugated and the cell pellets were stained with 2 μl 50ug/ml PI and 1 μl 100μg/mL RNase A for 30 min at 4°C in darkness. Subsequently, cells were analyzed by Flow cytometry. For cell apoptosis assay, the digested cells were stained with Annexin V-APC Apoptosis Detection Kit according to the manufacturer’s protocols (KeyGEN, Nanjing, Jiangsu, China). Then the apoptosis rates were detected using a flow cytometer.

### Migration Assay

Wound healing and Transwell experiments were employed to determine the migration ability of MDA-MB-231 cells. For wound healing assay, a linear wound was sketched with the pipette tip after cells reached a confluency of 90%-95%. Then cells were incubated in serum-free medium for 24 h and 48 h. Cells were then photographed at different time points and the migration rate was calculated by measuring the wound area. The wound areas at 0 h and 48 h were calculated by Image Pro Plus software. The healing rate was calculated using the formula: [wound area (0 h) - wound area (48 h)]/wound area (0 h). The transwell assay was also used to assess migration ability. Cells were plated into Transwell upper chamber at a density of 1x10^5^ cells/chamber (Millipore, Germany), and cultured with fresh medium without FBS. The lower chamber was surrounded with 600 μl of 10% FBS. After incubation for 12 h, cells on the upper chamber were wiped off and fixed for 30 minutes with ethanol. Then cells were stained with 0.1% crystal violet for 20 minutes after washing with PBS buffer for twice. Then, the migrated cells was calculated using a microscope (OLYMP^®^S, IX51, Japan).

### Quantitative Real-Time PCR

Total RNA was isolated utilizing TRIzol reagent (Roche, Indianapolis, IN, USA) according to manufacturer’s protocol. 1 μg total RNA was reversely transcribed into cDNA with All-in-One First-Strand cDNA Synthesis Kit (Transgene, China). Then, quantitative real-time PCR was performed using the SYBR Green kit (Roche, Indianapolis, IN, USA). GAPDH was served as the internal control. Relative gene levels were calculated using the 2^–ΔΔCt^ method.

### Western Blotting

Total protein was extracted from MDA-MB-231 and MCF-7 cells using RIPA buffer. Primary antibodies against extracellular signal-regulated kinase (ERK)1/2 and c-Myc were obtained from Santa Cruz Biotechnology (California, USA). Phosphorylated ERK1/2 and c-Jun N-terminal kinase (JNK)/p-JNK antibodies were purchased from Cell Signaling Technology (Boston, USA). ST5 antibody was purchased from Abcam BioTech. GAPDH (Sangon Biotech, Shanghai, Chian) and Tublin were served as the internal control. Grey scale intensity of the immunoblots was analyzed using Quantity One analysis software (Bio-Rad, California, US).

### DNA Methylation Determination

Bisulphite conversion of DNA samples from MDA-MB-231 cells was performed using the EZ DNA Methylation™ Gold Kit (ZYMO RESEARCH, USA) according to the manufacturer’ s instructions. After that, 2 μL of the eluted DNA was subjected to PCR under the following conditions: 98°C for 10 min, 53°C for 30 min, 53°C for 6 min, followed by 8 cycles at 37°C for 30 min, and 4°C storage. The primer sequences for methylation-specific PCR (MSP) were presented in **Table S1**. Then, the reaction products were detected by agarose gel electrophoresis.

### Statistical Analyses

All experiments were independently performed at least three times using independent samples. The number of ST5-positive cells and migrated cells were measured on the basis of at least five horizons by three independent researchers. Quantitative analysis of western blotting data, wound healing rate and apoptotic rate were performed using the GraphPad Prism 6.0 software with a two-tailed Student’s *t*-test. MTT assay data was analyzed by GraphPad Prism using two-way analysis of variance (ANOVA). Statistical analysis of clinical correlation was performed by the Cochran-Mantel-Haenszel and Chi-squared tests. Values have been presented as mean ± standard error of mean (SEM). Statistically significant differences were received at P < 0.05.

## Results

### ST5 Is Reduced in Breast Cancer Specimens

**Figure 1:**
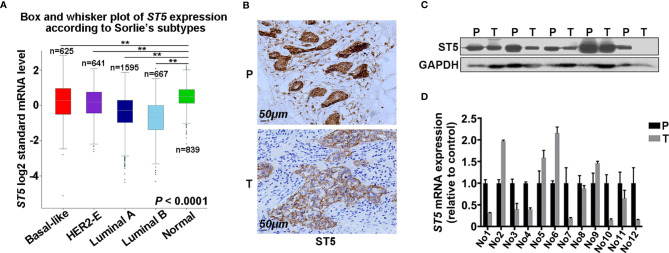
Evaluation of ST5 expression in human breast cancer specimens. **(A)** *ST5* gene expression as analyzed by using the bc-GenExMiner in different subtypes of human breast cancer tissues and normal breast tissues (n=839). The subtypes of human breast cancer included Luminal B subtype (n=667), Luminal A subtype (n=1,595), receptor tyrosine-protein kinase erbB-2 (HER2)-E subtype (n=641) and Basal-like subtype (n=625). ** indicated different subtypes of breast cancer vs. normal. **(B)** Immunohistochemical staining of ST5 in human breast cancer samples and its corresponding adjacent tissues. Scale bar: 50μm. Protein expression (n=5) **(C)** and mRNA level (n=12) **(D)** of ST5 in human breast cancer tissues and its paired para-carcinoma tissues were measured by Western blotting and qRT-PCR, respectively. P, para-carcinoma tissues; T, tumor tissues. **P < 0.01.

To investigate the role of ST5 in breast cancer, *ST5* gene expression was firstly analyzed in different subtypes of human breast cancer tissue and normal breast tissue using the bc-GenExMiner. Results demonstrated a lower level of *ST5* expression in multiple molecular subtypes of breast cancer tissue, including Luminal B subtype (n=667), Luminal A subtype (n=1,595), HER2-E subtype (n=641) and Basal-like subtype (n=625), than in normal breast tissue (n=839) (P < 0.01) (**Figure 1A**). Based on the immunohistochemical staining (IHC) results, a higher expression of ST5 in para-carcinoma tissues (P) than in paired tumor tissues (T) was observed (**Figure 1B**). After assessing the difference in protein level of ST5 between human breast cancer tissues and their corresponding normal breast tissues, results showed that ST5 level was observably declined in tumor samples (T) compared to that in paired normal breast tissue samples (P) (**Figure 1C**). Among these clinical tissue samples (n=12), mRNA expression approximately 58% of the tumor tissues (7/12) presented extremely low level of *ST5* mRNA as compared with their matching normal tissues (**Figure 1D**). Taken together, these findings demonstrate that ST5 status is decreased in human breast cancer specimens.

### ST5 Is Lowly Expressed in Invasive Breast Cancer

**Figure 2:**
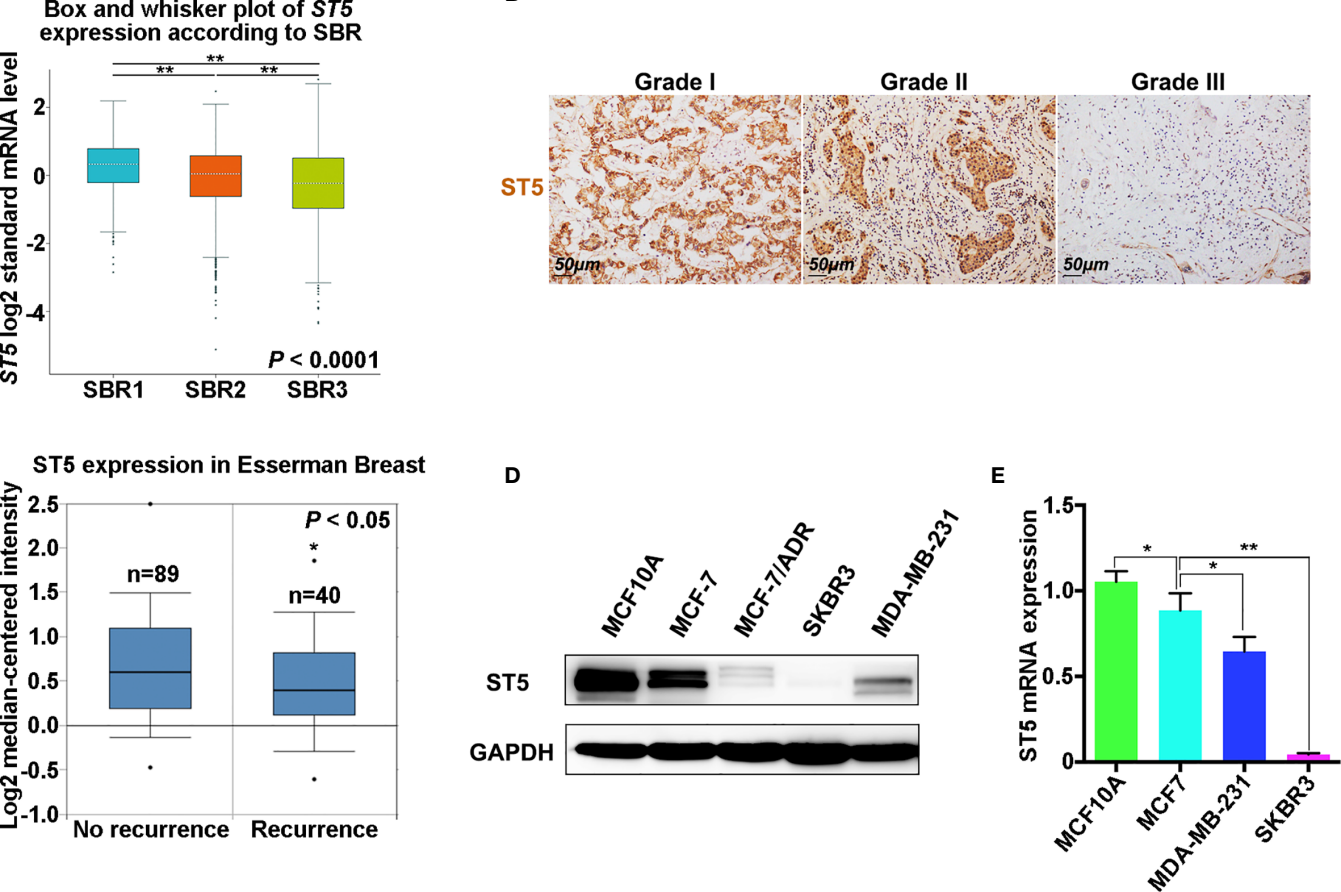
Expression analysis of ST5 in different breast cancer specimens and cells. **(A)** Analysis of ST5 expression status in different tumor grades according to Scarff Bloom and Richardson (SBR) classification as determined by using bc-GenExMiner v4.4. ** indicated SBR1 or SBR3 vs. SBR2 and SBR3 vs. SBR1. **(B)** Immunohistochemical staining using ST5 primary antibody in different pathological grades (Grade I, II and III) of human breast cancer tissue (tissue microarray). Scale bar, 50 μm. The brown areas indicated ST5-positive cells. **(C)** Differential expression of ST5 in non-recurrent or recurrent breast cancer patients in Esserman Breast using Oncomine database. * indicated Recurrence vs. No recurrence. **(D)** Protein level of ST5 in MCF-7, MCF-7/ADR, MDA-MB-231, SKBR3 cells and normal breast cells (MCF-10A) were determined by Western Blotting. MCF-10A, normal mammary epithelial cell line; MCF-7, ER-positive breast cancer cell; MCF-7/ADR, adriamycin resistant MCF-7 cells; MDA-MB-231, ER-negative cell line; SKBR3, ER/PR-positive and Her2-negative breast cancer cell lines. **(E)** Transcriptional level of ST5 in MCF-7, MDA-MB-231, SKBR3 cells and MCF-10A were determined by qRT-PCR. * indicated MCF-7 vs. MCF-10A and MDA-MB-231 or SKBR3 *vs*. MCF-7. *P < 0.05; **P < 0.01.

Next, the correlation between the expression of ST5 and tumor stage (SBR standard classification) was analyzed. Combined with the results from the bc-GenExMiner v4.4 database, a significant negative correlation between *ST5* status and tumor stage was verified, where patients with a higher tumor stage (SBR3) had lower *ST5* expression (**Figure 2A**). Uniformly, in the results of IHC assay, patients with advanced grade (grade II and grade III) always presented low ST5 expression compared to patients with early grade tumors (grade I) (**Figure 2B** **)**. Based on the staining intensity of ST5 using microarray, 33 out of 58 breast cancer patients presented low protein level of ST5 (- and +) and 26 out of 58 presented high level of ST5 (++ and +++). Correlation analysis indicated that ST5 status had no correlation with the clinicopathological characteristics of patients, including sex, age and tumor diameter. However, patients with advanced grade (grade 2: G2 and grade 3: G3) always showed low ST5 expression compared to patients with early grade tumors (G1) (P = 0.0397) (**Table 1**). Additionally, in comparison to patients with no recurrence (n=89), lower ST5 was also authenticated in recurrent breast cancer patients (**Figure 2C** **)**. In addition to that, based on the Oncomine database, there showed a notable decline in ST5 status in invasive ductal/lobular breast carcinoma (n=3 and 105) as compared to non-invasive ductal/lobular breast cancer patients (n=105 and 9) (**Figures S1A, B**). In the database of Nikolsky Breast, the decrease of ST5 copy number was also observed in invasive ductal breast carcinoma (n=5) by contrast to non-invasive ductal breast cancer specimens (n=133) (**Figure S1C**). These phenotypes also were confirmed in *in vitro* assays. As shown in **Figure 2D**, ST5 level was very low in breast cancer cells, such as MCF-7/ADR, MDA-MB-231 and SKBR3 cells compared to that in normal breast cells (MCF-10A), and ST5 status showed more lower in cell lines with a high invasive capability (SKBR3 and MDA-MB-231) (**Figure 2D**). Moreover, the mRNA expression of *ST5* was notably decreased in breast cancer cells compared to that in normal MCF-10A cells, indicating lower ST5 levels in invasive cell lines (MDA-MB-231: a 50% fall; SKBR3: a 85% fall, P = 0.0001) than those in MCF-7 cells with a lower invasive capability (a 25% fall) (**Figure 2E**). The data indicate low level of ST5 may most likely be observed in invasive breast cancer specimens.

### ST5 Affects Cell Viability and Migration of Invasive Breast Cancer Cells

**Figure 3:**
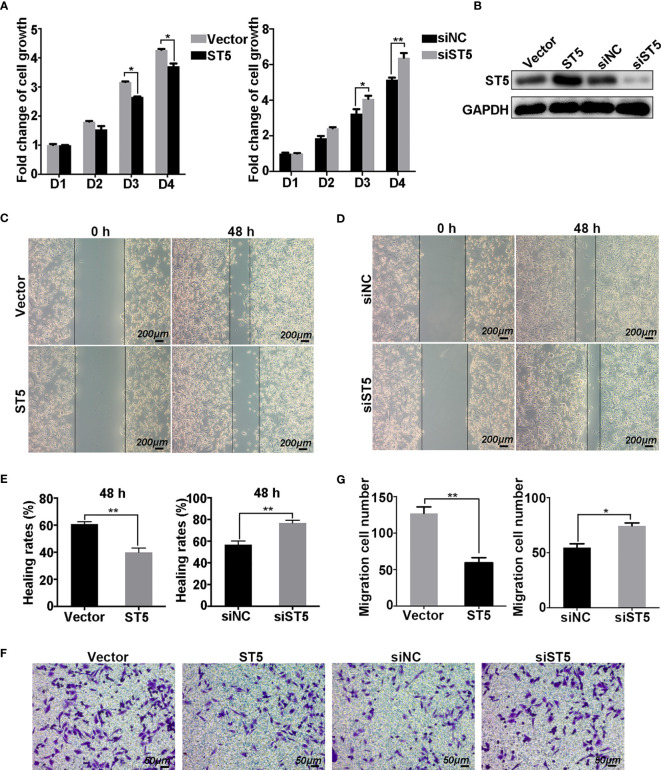
Effect of ST5 on cell proliferation and migration of MDA-MB-231 cells. MTT assay was performed to measure the growth ability of MDA-MB-231 cells transfected with control plasmid (Vector) and ST5-overexpressing plasmid (ST5) and MDA-MB-231 cells transfected with negative control siRNA (siNC) and siRNA targeting ST5 (siST5) **(A)**. **(B)** The verification of transfection efficiency of ST5 overexpressing plasmid or knockdown siRNA as detected by Western Blotting. Images of wound healing in Vector and ST5 group **(C)**, siNC and siST5 group **(D)** before and after wound healing for 48 h Scale bar, 200μm. **(E)** Healing rates in MDA-MB-231 cells transfected with Vector, ST5, siNC or siST5 as analysed by using Image Pro Plus software (version 6.0). **(F)** Cell migration was determined using Transwell assay and the migrated cells were stained by 0.1% crystal violet for 20 minutes. Scale bar, 50μm. **(G)** The number of cells migrated to the lower chamber as calculated by using Image Pro Plus software. * indicated cells transfected with ST5-overexpressing plasmid or siST5 *vs*. Vector or siNC, respectively. *P < 0.05; **P < 0.01.

To assess the cellular functions of ST5, cell proliferation and migration assays were performed in MDA-MB-231 (invasive cell line) and MCF-7 cells (cells with a lower invasive capability) transfected with exogenetic ST5 and siRNAs targeting ST5. Results showed a robust suppression of cell viability in the ST5 overexpressing group compared with that in the control vector group from days 3 to 4 (**Figure 3A**), while RNAi-mediated knockdown of ST5 resulted in the upregulation of cell viability on day 3 (P < 0.05), and day 4 (P < 0.01) in the ST5-knockdown group compared to that in the cells transfected with negative control siRNA group (**Figure 3A**). The expression of ST5 in ST5 overexpressing or ST5-knockdown cells were verified by WB (**Figure 3B**). In the Wound Healing experiment, according to the changes of wound area, the healing rate notably reduced by 21% at 48 h (P < 0.01) post-wounding in the ST5 group compared to that in the vector group (**Figures 3C, E** **)**. Moreover, ST5 depletion increased by 20% at 48 h (P < 0.01) in wound healing rate as compared to that in the control cells (**Figures 3D, E** **)**. Additionally, in the Transwell assay, a 50% reduction (P < 0.01) in the number of migratory cells following overexpression of ST5, in contrast to a 30% increase (P < 0.05) in the number of migratory cells observed in ST5-depleted cells, as compared with the corresponding control cells (**Figures 3F, G**). However, forced expression of ST5 or ablation of ST5 had no effect on cell viability and migration in MCF-7 cells compared to the corresponding control cells (**Figures S2A, D**). Thus, these results indicate a potential anti-neoplastic effects of ST5 on invasive breast cancer cells.

### ST5 Induces Cell Cycle Arrest and Apoptosis in Invasive Breast Cancer Cells

**Figure 4:**
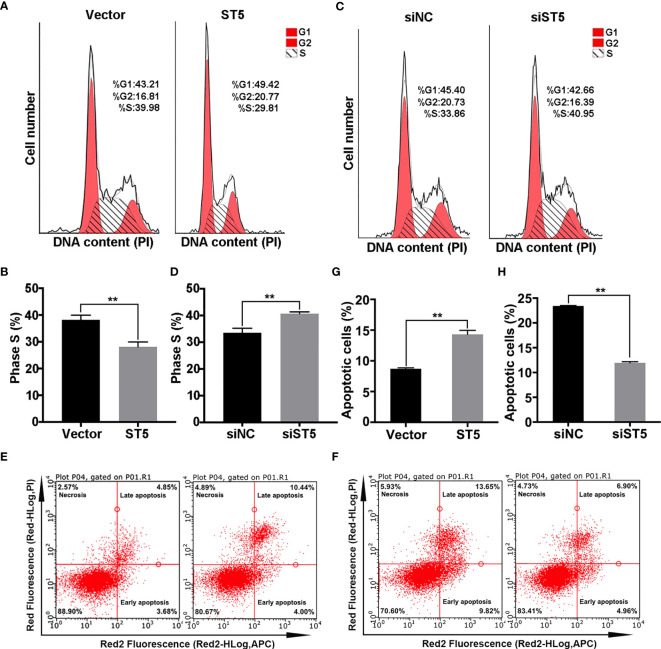
Effect of ST5 on cell cycle and apoptosis of MDA-MB-231 cells. **(A)** Cell cycle progression was analysed in MDA-MB-231 cells transfected with Vector and ST5 plasmids. **(B)** Quantitative analysis of the proportion of phase S cells in panels **(A, C)** Cell cycle progression was analysed in MDA-MB-231 cells transfected with negative control siRNA and ST5-siRNA (siST5). **(D)** Quantitative analysis of the proportion of phase S cells in panel **(C)** The effect of ST5-upregulation on cell apoptosis **(E)** or effect of ST5-downregulation on cell apoptosis **(F)** were analysed by flow cytometry. Quantitative analysis of the proportion of apoptotic cells in the vector and ST5 groups **(G)** and in the siNC and siWDR41 groups **(H)** was presented. * indicated cells transfected with ST5-overexpressing plasmid or siST5 *vs*. Vector or siNC, respectively. *P < 0.05, **P < 0.01.

Next, flow cytometry analysis was employed to determine the impact of ST5 on cell cycle and programmed cell death. As shown in **Figure 4A**, forced expression of ST5 in MDA-MB-231 cells decreased the number of S phase cells by 10% (P < 0.01) (**Figures 4A, B**), while ST5 ablation increased the proportion of S phase cells by 7% (P < 0.01) compared to that in siNC group (**Figures 4C, D**). Based on the apoptosis assay, approximately a 5.6% increase in the proportion of apoptotic cells was observed in (P < 0.01) in ST5-overexpressing cells (**Figures 4E, G** **)**. Conversely, knockdown of ST5 markedly reduced the proportion of apoptotic cells by 11.5% (P < 0.01) (**Figures 4F, H** **)**. In MCF-7 cells, either ST5 upregulation or downregulation did not affect cell cycle progression (**Figures S3A, B**) and programmed cell death (**Figures S3C–F**). Accordingly, our data demonstrate that ST5 moderately abrogates the DNA synthesis, resulting in cell cycle arrest and a subsequent increase in apoptosis.

### ST5 Inactivates the Activation of ERK/JNK Signaling Pathway

**Figure 5:**
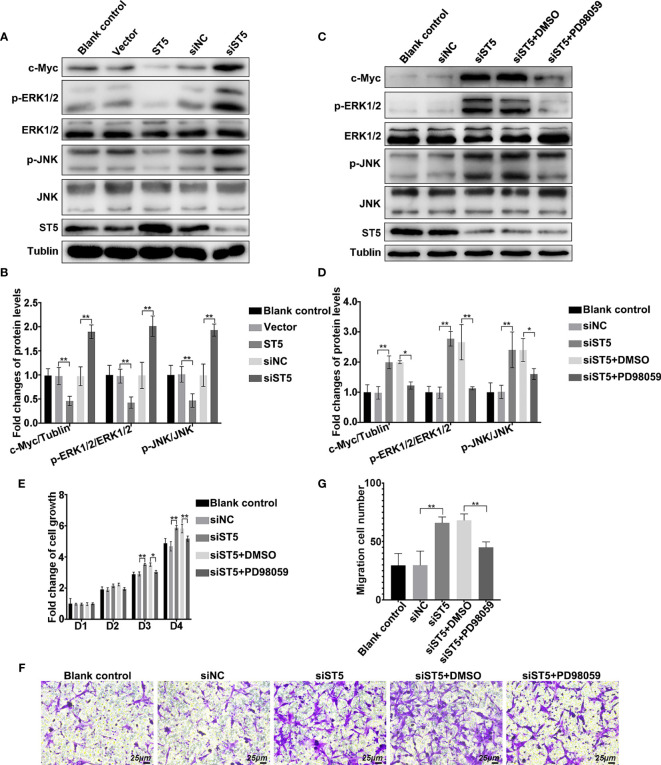
Regulatory mechanism of ST5-mediated tumour inhibition. **(A)** The activation of extracellular signal-regulated kinase (ERK)1/2 and c-Jun N-terminal kinase (JNK), and the expression of c-Myc and ST5 were detected by western blotting in ST5-depleted and ST5-overexpressed MDA-MB-231 cells. **(B)** Relative quantification of phosphorylated ERK1/2 and JNK, and c-Myc expression in panel **(A)** * indicated cells transfected with ST5-overexpressing plasmid or siST5 vs. Vector or siNC, respectively. **(C)** MDA-MB-231 cells were transfected with siRNA of ST5 alone or the combination of siRNA of ST5 and 10 μM PD98059. The activation of ERK1/2 and JNK, and the expression of c-Myc were detected by western blotting after upregulation of ST5 in MDA-MB-231 cells. **(D)** Relative quantification of phosphorylated ERK1/2 and JNK and c-Myc in panel **(C, E)** MDA-MB-231 cells were transfected with siRNA of ST5 alone or the combination of ST5-siRNA and 10 μM PD98059. Subsequently, MTT assay was performed to measure the cell viability. **(F)** MDA-MB-231 cells were transfected with ST5-siRNA or the combination of ST5-siRNA and 10 μM PD98059. Cell migration was determined using Transwell assay and the migrated cells were stained by 0.1% crystal violet for 20 minutes. Scale bar, 25μm. **(G)** The number of cells migrated to the lower chamber as analysed by using Image Pro Plus software. *, siST5 or siST5+PD98059 *vs*. siNC or siST5+DMSO, respectively. *P < 0.05, **P < 0.01.

Consequently, the signaling cascade affected by ST5 in invasive breast cancer cells was verified. As shown in **Figure 5**, overexpression of ST5 restrained the phosphorylation of ERK1/2 and JNK, and decreased the expression c-Myc (**Figures 5A, B**). However, ablation of ST5 significantly promoted the activation of ERK1/2 and JNK, and the expression of c-Myc (**Figures 5A, B**). Of note, ST5 downregulation-mediated the activation of MAP kinases ERK and JNK, and the elevation of c-Myc protein, were notably depressed in the presence of PD98059, an inhibitor of MEK (ERK1/2) (**Figures 5C, D**). The exposure of PD98059 did not affect the level of ST5 (**Figure 5C**). To make sure that the ERK pathway mediated the effect of ST5 on cancer progression, MTT and Transwell experiments were also conducted in ST5-depleted cells under the condition of PD98059. As shown in **Figure 5E**, RNAi-mediated knockdown of ST5 significantly reduced cell viability on day 3 (P < 0.01), and day 4 (P < 0.01) in the ST5-knockdown group compared to that in the cells transfected with negative control siRNA group (**Figure 5E**), while PD98059 addition in ST5-knockdown group notably abrogated ST5 knockdown-mediated cell over-proliferation on day 3 (P < 0.05), and day 4 (P < 0.01) (**Figure 5E**). Additionally, a 50% increase (P < 0.01) in the number of migratory cells ST5-depleted cells, whereas a 32% reduction (P < 0.01) in the number of migratory cells following the challenge of PD98059 in ST5-depleted cells, in contrast to that observed in ST5-depleted cells (**Figures 5F, G**). Overall, ERK/JNK signaling pathway was negatively regulated by ST5 in invasive breast cancer cells. Possibly, the potential anti-tumor effects of ST5 on invasive breast cancer cells depends on ERK/JNK signaling pathway.

### Hypermethylation of ST5 Promoter Region Affects Cell Viability and Migration of Invasive Breast Cancer Cells

**Figure 6:**
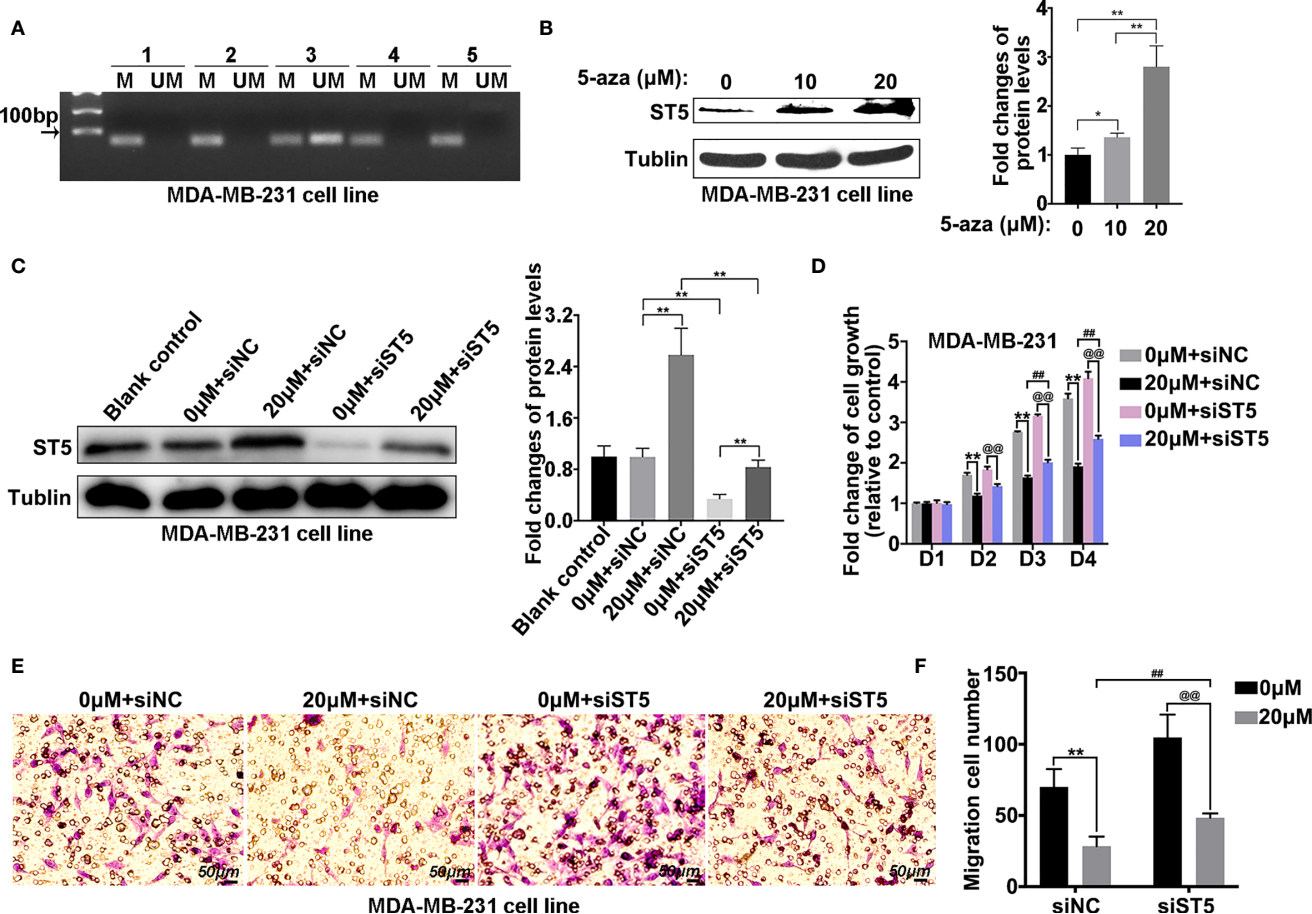
Methylation level of the ST5 promoter region in MDA-MB-231 cells. **(A)** Methylation-specific PCR (MSP) was performed to measure the methylation level of ST5 in MDA-MB-231 cells. The number (one to five) indicated different primers used in methylation detection. **(B)** Protein expression of ST5 was evaluated by WB in MDA-MB-231 cells after treatment with different doses of 5-aza-dC or DMSO for 72 **(h)** Relative quantification of ST5 in 5-aza-dC-challenged cells as presented in the right. **(C)** MDA-MB-231 cells were exposed to 0 μM or 20 μM 5-aza-dC for 48 **(h)** Subsequently, 0 μM or 20 μM 5-aza-dC-challenged cells were transfected with negative control siRNA (siNC) or ST5-siRNA. Protein expression of ST5 as determined by WB. Relative quantification of ST5 as presented in the right. **(D)** MDA-MB-231 cells were transfected with negative control siRNA and siRNA targeting ST5. Subsequently, both cells were treated with 20 μM 5-aza-dC or DMSO for 72 h MTT assay was performed to measure the cell viability of MDA-MB-231 cells. **(E)** Transwell assay was performed to measure the cell migration of MDA-MB-231 cells treated with/without 20 μM 5-aza-dC in cells transfected with negative control siRNA and ST5-siRNA. **(F)** The number of migration cells as calculated by using Image Pro Plus software. M, methylation primer; UM, unmethylated primer. * indicated 20 μM 5-aza-dC-treated control cells vs. DMSO-treated control cells; # indicated 20 μM 5-aza-dC-treated siST5 group vs. 20 μM 5-aza-dC-treated control group; @ indicated 20 μM 5-aza-dC-treated siST5 group vs. DMSO-treated siST5 group. **, ##, @@, P < 0.01.

Gene expression is always regulated by epigenetic changes. Then, the methylation level of the ST5 promoter region in MDA-MB-231 cells was determined by methylation-specific PCR with five pairs of primers that were designed using the MethPrimer method (**Table S1**). MSP data indicated that hypermethylation of ST5 promoter was found in the MDA-MB-231 cells (**Figure 6A**). To investigate whether promoter methylation of ST5 affected its protein expression, the protein level of ST5 in breast cancer cells was detected using 5-aza-dC, an inhibitor of DNA methylation. An increase in 5-aza-dC dosage (10 μM and 20 μM) affected the expression of ST5 in MDA-MB-231 cells (**Figure 6B**). As shown in **Figure 6C**, 5-aza-dC-challenged cells appeared an increase of ST5 expression, while ST5 siRNA transfection notably reduced the expression of ST5 in 5-aza-dC-exposed cells (**Figure 6C**). Interestingly, the cell viability of MDA-MB-231 cells were inhibited by the exposure of 20 μM methylation inhibitor from day 2 to day 4. By contrast, once ST5 was knocked down, 5-aza-dC-mediated proliferation inhibition was notably reversed on days 3 and 4 (**Figure 6D**). In the Transwell assay, a 60% reduction (P < 0.001) in migration cell number following 5-aza-dC treatment was observed, as compared with the corresponding control cells. However, under the treatment of 5-aza-dC, the number of migration cells in ST5-depleted group was notably higher than that in negative control-transfected cells (**Figures 6E, F**). Actually, 5-aza-DC treatment appeared to demethylate many genes including ST5, resulting in the elevation of ST5 and other protein levels. But, 5-aza-dC-mediated the increase of ST5 protein, following by the proliferation inhibition, was significantly abolished by siRNA-mediated the downregulation of ST5 protein. Possibly, promoter methylation-mediated the decline of ST5 was involved in the progression of tumor metastasis in breast cancer cells.

## Discussion

Currently, a combination of surgery and chemotherapy or radiation therapy is the most common therapeutic method in clinic for breast cancer; however, poor prognosis and relapse still occur frequently in patients with distant metastases (22). Although advanced targeted cancer therapy and neoadjuvant therapy are constantly emerging, the therapeutic strategies and effects for metastatic breast cancer are limited clinically (23–26). Therefore, it is of great significance to search for more specific molecular targets for treating metastatic breast cancer. ST5 shows differentially expression in tumorigenic and nontumorigenic somatic cell hybrids of Hela cells, implying ST5 may affect tumorigenesis (9). In the present study, ST5 was lowly expressed in invasive breast cancer. ST5 upregulation abolished tumor migration of breast cancer cells and hypermethylation-caused the decline of ST5 may implicate in the metastatic progress of breast cancer. Probably, ST5 acts as a potential therapeutic target for metastatic breast cancer.

ST5 is differential expression in several gynecological oncology (12, 14). The full length of ST5 (P126kD) robustly elevated in uterine leiomyoma as compared to normal myometrium (14). Here, the decline of full length ST5 (P126kD) was observed in human breast cancer, particularly in invasive breast cancer cells. However, the data does not detect the alterations of the other two variants of ST5 (P70 and P82) among these breast cell types. However, the finding firstly demonstrated that ST5 (P126kD) showed differential expression among normal mammary epithelial cells, non-invasive and invasive breast cancer cells. Interestingly, the high invasive capability cell line SKBR3, which is ER- and progesterone receptor (PR)-negative but HER2-positive, presented lower ST5 level than MDA-MB-231 cells. We suspected that the reason was as follows. On the basis of analysis results from bc-GenExMiner v4.4, ST5 status also appeared a significant decline in HER2-positive breast cancer patients (n=661) compared to that in HER2-negative patients (n=3582) (**Figure S4**). The data imply a potential negative association between HER2 and ST5 in breast carcinogenesis which still needs to study further. Additionally, in this study, ST5 expression status was not associated with clinical characteristics of breast cancer subjects including gender, age and tumor diameter, but was negatively associated with the tumor grade. The data demonstrated that ST5 was gradually declined with tumor progression of breast cancer. However, ST5 expression trend in different tumor grade of metastatic breast cancer requires further confirmation.

The cleavage ST5 (P70kD) decreases tumorigenicity in HeLa cells, implying it may serve as a cancer suppressor gene (13, 27). Additionally, ST5 (P70kD) is correlated with decreased tumorigenic phenotype in mammalian cells, with a restoration in their transformed phenotype and contact-dependent growth (9, 28). The C-terminal region of ST5 (P126kD) binds and catalyzes the exchange of GDP to GTP of the Rab13 protein, contributing to promote metastatic behavior (21). Based on our present data, full length of ST5 (P126kD) also possessed the inhibition effect on biological behavior of invasive breast tumor cells. Thus, full length ST5 plays the opposite effect between invasive breast cancer cells and normal breast epithelial cell. However, ST5 did not affect the tumor behaviors of MCF-7 cells. We speculated that the reason might be due to the presence of ER that was expressed in MCF-7 cells, but not in MDA-MB-231 cells. Whether overexpression of ST5-mediated antitumor effect is prevented by the presence of ER in MCF-7 cells remains unclear.

ST5 is important for cellular signal cascades and the regulation of the transformed phenotype (29). Structurally, ST5 contains a group of GDP/GTP exchange region for Rab protein family and the mitogen-activated protein (MAP) kinase-activating death region which is verified as a interactant of TNF-alpha receptor (30, 31). The shortest form ST5 (P70) is related with low tumorigenicity and the longest ST5 (P126) can activate the MAP kinase ERK1/2 in response to epidermal growth factor (EGF) in cos-7 African green monkey fibroblast cells (32). The proline-rich region of ST5 isoform can recognize Src homology 3 (SH3) binding domains and MAP kinase phosphorylation sites (33–35), and participate in RAS/MEK2/ERK2 signaling cascade (28). Additionally, DENN/MADD, a multifunctional domain protein, interacts with JNK, activating MAPK/JNK pathway in Alzheimer’s disease (AD) pathogenesis (36, 37). Therefore, ST5-mediated intracellular signaling transduction has the involvement of ERK1/2/JNK pathway. In the present study, we observed that ERK1/2/JNK activation was obviously inhibited, followed by the decrease of c-Myc in MDA-MB-231 cells transfected with exogenous ST5, vice versa. Of note, ERK1/2 inhibition not only abolished the increase of c-Myc and the activation of JNK in ST5-depleted cells, but also rescued the stimulation of cell proliferation and migration caused by ST5 knockdown. Possibly, ERK1/2/JNK signal axis was the downstream of ST5 during the migration process of breast tumor cells. However, whether ST5-mediated the inactivation of ERK1/2/JNK signals involved in the exchange of GDP to GTP of the Rab protein family which is the main feature during metastatic behavior of cancer cells, remains to be explored in the future study.

Tumor suppressors are always repressed by diverse of factors, such as microRNAs, transcription factors, and DNA methylation, followed by the decline of tumor suppressors, resulting in tumor progression (38, 39). Similar to other cancer types, human breast cancer also exhibits a high number of epigenetic alterations in the genome, particularly in tumor suppressors (40). Methylation profiles can be used as clinical biomarkers to predict drug response and prognosis of breast cancer (41, 42). Of note, DNA methylation of tumor suppressors have become the effective biomarker for early diagnosis and disease progression monitoring in the invasive breast tumors (43–45). Among the DENN protein family, promoter hypermethylation-mediated the downregulation of DENND2D is associated with early recurrence of hepatocellular carcinoma and gastric cancer (16, 17). The previous research from our research group has revealed hypermethylated ST5 promoter region in gastric poorly differentiated adenocarcinoma, appearing low expression of ST5 in advanced gastric cancer (46). Herein, promoter region of ST5 was proved highly methylated in invasive breast cancer cells. And methylation inhibitor not only promoted ST5 expression, but also rescued tumor characteristics of MDA-MB-231 cells. However, methylation inhibitor-induced the increase of ST5 protein and the proliferation inhibition were partially reversed by siRNA-mediated the decline of ST5. Interestingly, knockdown of ST5 didn’t play a particularly significant acceleration role on cell viability in 5-aza-DC-challenged cells. It’s probably because 5-aza-DC appeared to demethylate many tumor suppressor genes, leading to decline sharply in cell viability. And the depletion of ST5 did not cover the anti-proliferative effects induced by the activation of other tumor suppressor genes. Possibly, epigenetic modification activating the expression of ST5 may be used as a potential therapeutic target for metastatic breast cancer.

The complexity of epigenetic modifications should not make us neglect the fact that other epigenetic regulatory mechanisms can also implicate in the reduction of ST5 in invasive breast cancer. Additionally, in comparison to other tumor suppressor, ST5 only mediates anti-tumor and anti-migration effects in TNBC cells with a high-invasive capability, but not in MCF-7 cells with a low-invasive capability. Possibly, the presence of ER, PR or HER2 prevents ST5-mediated the exchange of GDP to GTP in Rab protein family, resulting in the negative feedback of ST5 on tumor behaviors in MCF-7 cells.

## Conclusions

In summary, we elaborated the anti-tumor role of ST5 in invasive breast cancer cells through regulating ERK1/2/JNK signaling pathway. Abnormal epigenetic modification causes the decline of ST5 expression, subsequently promoting breast tumor cell metastasis. Possibly, targeting epigenetic mechanisms and/or exogenous ST5 contribute to blocking the proliferation and the metastasis of breast cancer and may be used as a promising advanced therapeutic method for metastatic breast cancer.

## Data Availability Statement

The original contributions presented in the study are included in the article/**Supplementary Material**. Further inquiries can be directed to the corresponding authors.

## Ethics Statement

Written informed consent was obtained from the individual(s) for the publication of any potentially identifiable images or data included in this article.

## Author Contributions

Conceptualization, SC and XG. Methodology, JC. Software, SC. Validation, JC and ML. Formal Analysis, JC. Investigation, CT. Resources, SC and XG. Data Curation, CT. Writing – Original Draft Preparation, SC. Writing – Review and Editing, CT. Visualization, JC. Supervision, XG. Project Administration, SC. Funding Acquisition, SC, XG and JC.

## Funding

This work was supported by Natural Science Foundation of China (81802332), Natural Science Foundation of Fujian Province (2020J05302), Natural Science Basic Research Plan in Shaanxi Province of China (2021JQ-780 and 2020JQ-879), Research Foundation of Xi’an Medical University (2017DOC18 and 2018GJFY04) and Young Talent Fund of University Association for Science and Technology in Shaanxi (20190309).

## Conflict of Interest

The authors declare that the research was conducted in the absence of any commercial or financial relationships that could be construed as a potential conflict of interest.

## Abbreviations

ST5, Suppression of tumorigenicity 5; MSP, methylation-specific PCR; ER, estrogen receptor; PR, progesterone receptor; HER2, receptor tyrosine-protein kinase erbB-2; TNBC, triple-negative breast cancer; MAP, mitogen-activated protein; JNK, c-Jun N-terminal kinase; AD, Alzheimer’s disease.
