# Supplementary figures and images for: Suppression of Tumorigenicity 5 Ameliorates Tumor Characteristics of Invasive Breast Cancer Cells via ERK/JNK Pathway
Source: Front Oncol. 2021 Jul 28;11:621500. doi: 10.3389/fonc.2021.621500 (PMC8356645; doi:10.3389/fonc.2021.621500)

Raw data for blots

Figure 3B

ST5

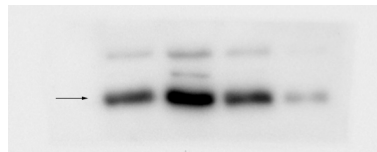

GAPDH

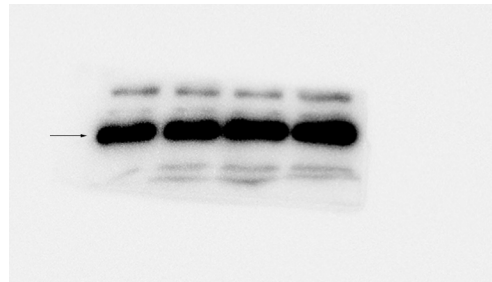

Figure 5A

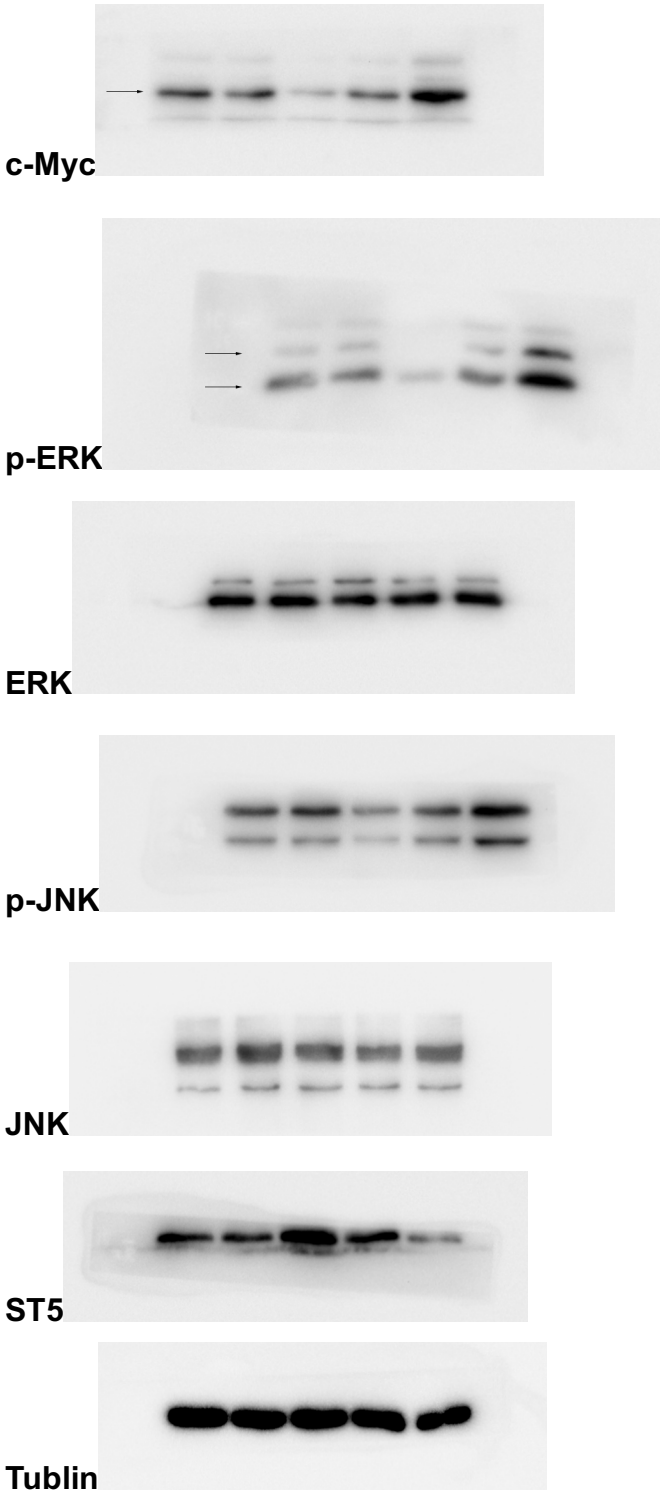

Figure 5C

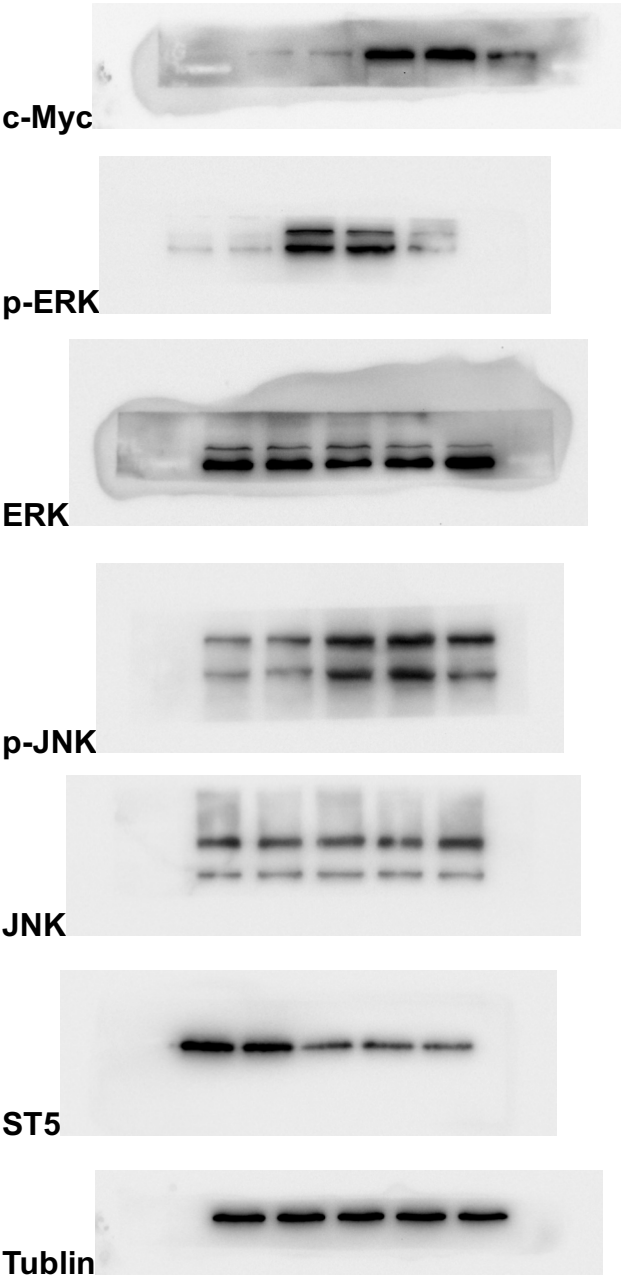

Figure 6B

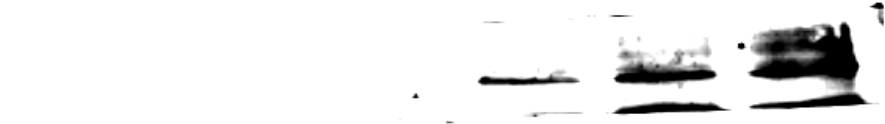

ST5

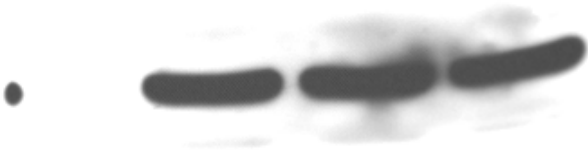

Tublin

**Figure 6C**

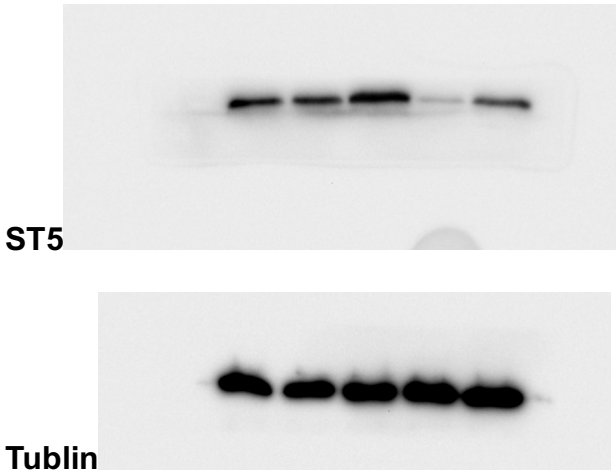

Figure S2

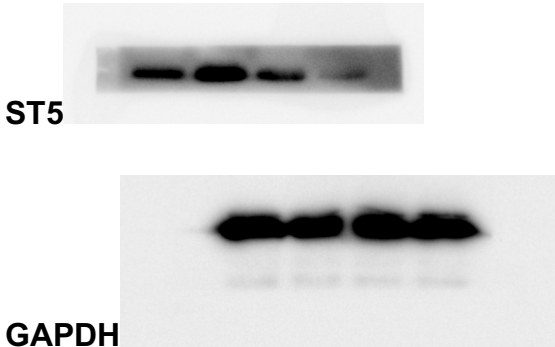

Supplement: Supplementary file 1 [file DataSheet_1.pdf]
