# Supplementary material for: Suppression of Tumorigenicity 5 Ameliorates Tumor Characteristics of Invasive Breast Cancer Cells via ERK/JNK Pathway
Source: Front Oncol. 2021 Jul 28;11:621500. doi: 10.3389/fonc.2021.621500 (PMC8356645; doi:10.3389/fonc.2021.621500)
Supplement: Supplementary file 2 [file DataSheet_2.pdf]

Supplementary Material

1 Supplementary Figures and Tables

1.1 Supplementary Figures

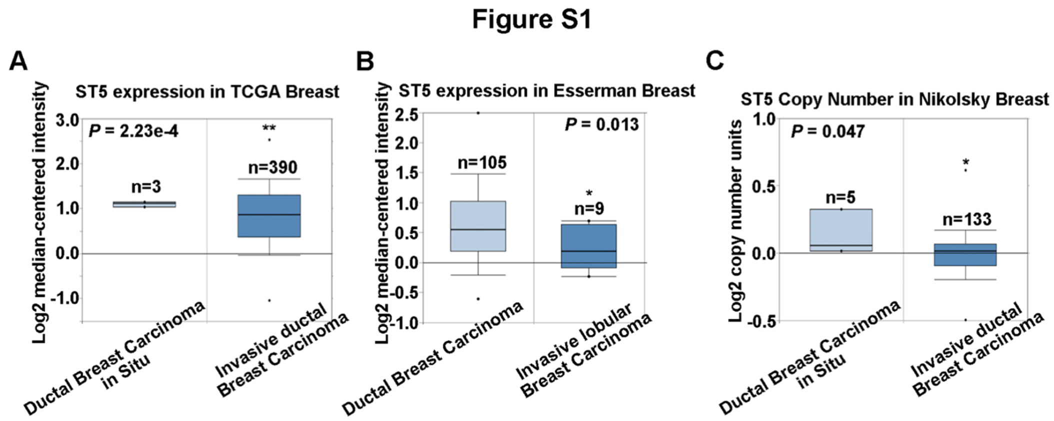

Supplementary Figure 1. Analysis of ST5 differential expression in invasive and non-invasive breast cancer. ST5 differential expression between invasive ductal breast carcinoma and non-invasive ductal/lobular breast cancer patients in TCGA breast database (A), and between invasive lobular breast carcinoma and non-invasive lobular breast cancer patients in Esserman breast database (B). (C) The difference in ST5 copy number between invasive ductal breast carcinoma and non-invasive

ductal breast cancer specimens. * indicated invasive lobular/ductal breast carcinoma vs. ductal breast cancer specimens. *, P < 0.05; **, P < 0.01.

Figure S2

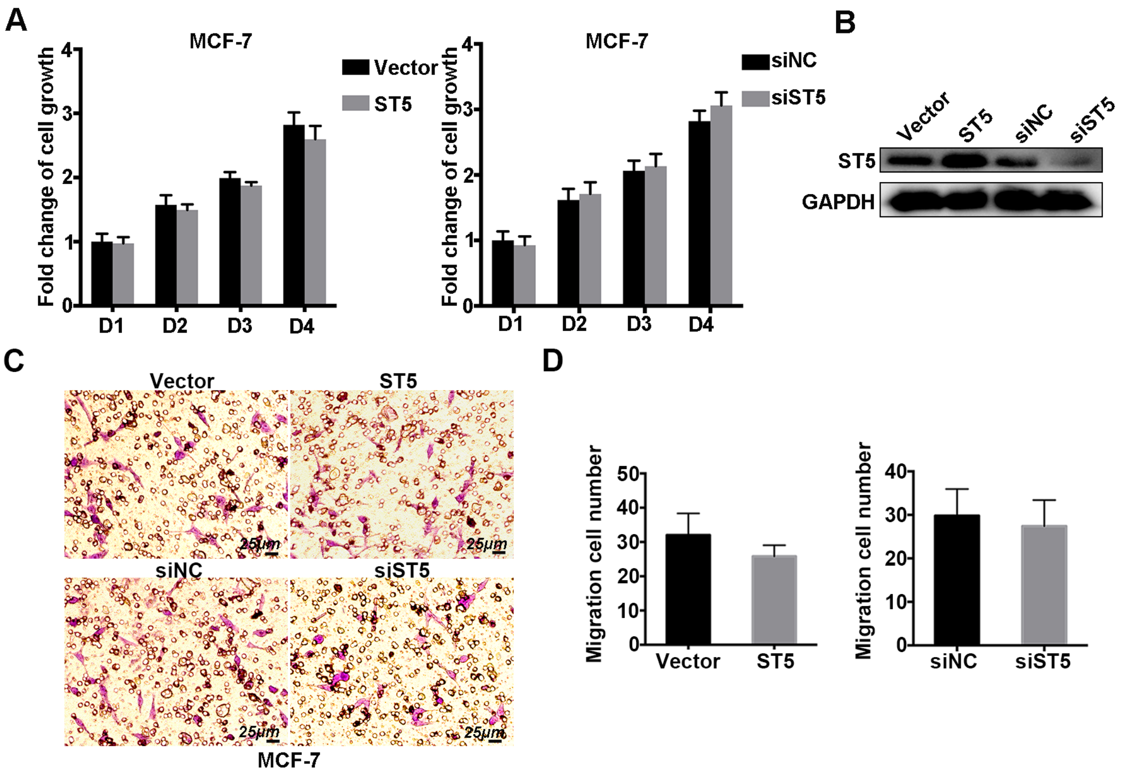

Supplementary Figure 2. Effect of ST5 on cell proliferation and migration of MCF-7 cells. MTT assay was performed to measure the cell growth ability of MCF-7 cells transfected with control plasmid (Vector) and ST5-overexpressing plasmid (ST5) and cells transfected with negative control siRNA (siNC) and siRNA targeting ST5 (siST5) (A). The verification of transfection efficiency as detected by Western Blotting and showed above the corresponding histograms (B). (C) Cell migration was determined using Transwell assay and the migration cells were stained by crystal violet. Scale bar, 25μm. (D) The number of migration cells as calculated by using Image Pro Plus software.

Figure S3

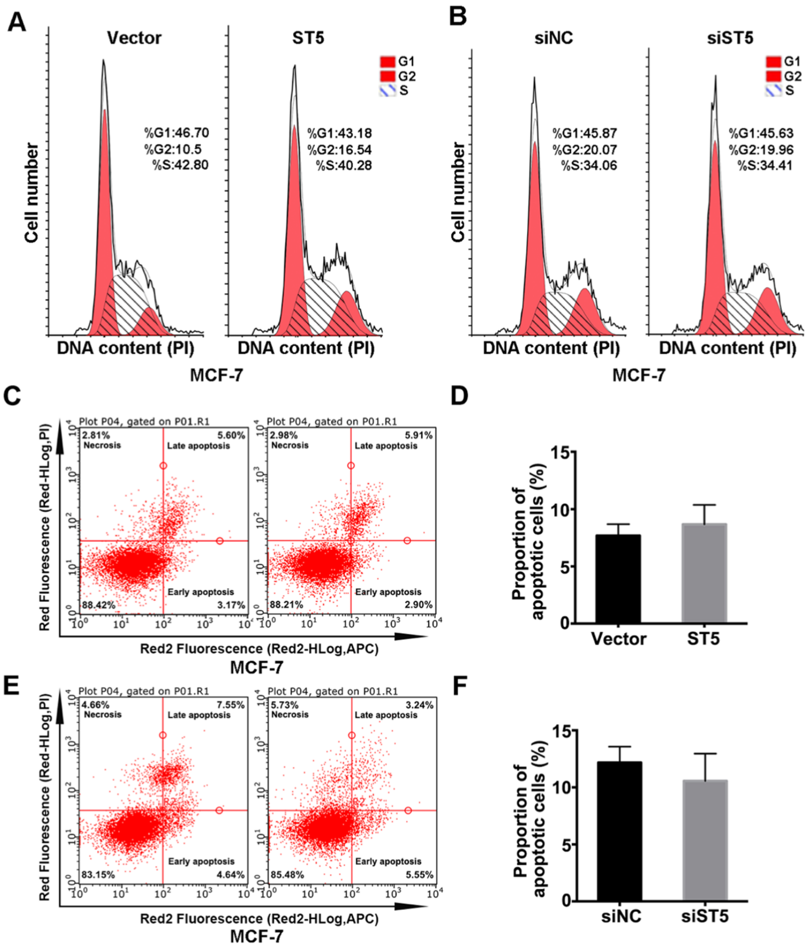

Supplementary Figure 3. Effect of ST5 on cell cycle and apoptosis of MCF-7 cells. Cell cycle progression was analysed in MCF-7 cells transfected with vector and ST5 plasmids (A) or transfected with negative control siRNA and ST5-siRNA (B). (C) The effect of ST5-upregulation on cell apoptosis was analysed by flow cytometry. (D) The proportion of apoptotic cells was measured in the vector and ST5 groups. (E) The effect of ST5-downregulation on cell apoptosis was analysed by flow cytometry. (F) The proportion of apoptotic cells was measured in the siNC and siST5 groups.

Figure S4

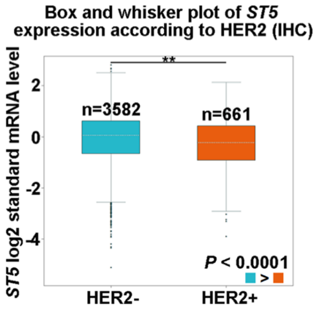

Supplementary Figure 4. Analysis of ST5 differential expression in HER2-negative and HER2-positive breast cancer specimens. The differential expression of ST5 in HER2-negative (n=3582) and HER2-positive (n=661) breast cancer specimens as analysed by bc-GenExMiner v4.4. **, $P < 0.01$.

1.2 Supplementary Tables

Table S1 Primers for methylation specific PCR

Gene	Left M Primer	Right M Primer	Left U Primer	Right U Primer
ST5-1	GTTTTTATGATAATCGGTTTGGTC	CTTAAACTCCGATACCTACTACGAA	TTTTATGATAATTGGTTTGGTTGG	ACTTAAACTCCAATACCTACTACAAA
ST5-2	GTTTTTATGATAATCGGTTTGGTC	CTTAAACTCCGATACCTACTACGAA	TTTTATGATAATTGGTTTGGTTGG	CTTAAACTCCAATACCTACTACAAA
ST5-3	CGAGGATAGGGTTTTTATGATAATC	CTTAAACTCCGATACCTACTACGAA	TGAGGATAGGGTTTTTATGATAATTG	CACTAACTTAAACTCCAATACCTACTACAA
ST5-4	GTTTTTATGATAATCGGTTTGGTC	ACTTAAACTCCGATACCTACTACGAA	TTTTATGATAATTGGTTTGGTTGG	ACTTAAACTCCAATACCTACTACAAA
ST5-5	TTTTTATGATAATCGGTTTGGTC	CTTAAACTCCGATACCTACTACGAA	TTTTATGATAATTGGTTTGGTTGG	ACTTAAACTCCAATACCTACTACAAA
